# The Effects of Aloe Vera Cream on the Alar Scar in Rhinoplasty, A Randomized Double-Blind Controlled Trial

**DOI:** 10.22038/IJORL.2024.77572.3601

**Published:** 2024-05

**Authors:** Reza Kaboodkhani, Mohsen Sarikhani, Tayebeh Kazemi, Mohammad Mehdi Zarshenas, Mohammad Miaad Shahrizi, Maryam Sadat Sadati, Seyed Hossein Owji

**Affiliations:** 1 *Otolaryngology Research Center, Department of Otolaryngology, Shiraz University of Medical Sciences, Shiraz, Iran.*; 2 *Department of Phytopharmaceuticals (Traditional medicine), School of Pharmacy, Shiraz University of Medical sciences, Shiraz, Iran.*; 3 *Molecular Dermatology Research Center, Shiraz University of Medical Sciences.*; 4 *Student Research Committee, Shiraz University of Medical Sciences, Shiraz, Iran.*

**Keywords:** Aloe vera, Cicatrix, Wound healing, Rhinoplasty

## Abstract

**Introduction:**

Many studies have been done on the use of aloe vera in wound healing, but fewer studies were done on the influence of this material on the reduction of the alar scar. Therefore, we evaluated the effect of a newly made aloe vera cream on alar wound healing after rhinoplasty.

**Materials and Methods:**

This was a randomized, double-arm, parallel-group, double-blind controlled trial and was done from June 2021 to February 2022. External wedge resection was done for all patients. The patients were randomly assigned to receive aloe vera cream (n=31) (intervention group) or Face Doux cream (comparison group) (n = 29). A pharmacist prepared the aloe vera cream. The primary outcome measure was the wound scar status which was assessed by two Questionnaires, including the mean Patient Scar Assessment Questionnaire (PSAQ) and Vancouver Scar Scale (VSS). Randomization and Blinding were done.

**Results:**

The mean PSAQ was significantly lower in group A after two weeks (26.9 versus 31.5, P<0.001), after two months (15.7 versus 19.6, P=0.04), and six months follow-up (8.8 versus 11.8, P=0.005). The mean VSS was significantly lower in group A after two weeks (5.6 versus 7.1, P=0.001), after two months (3.5 versus 4.9, P=0.002), and six months (1.2 versus 2.7, P<0.001). Repeated measurement analysis showed that both interventions significantly affected PSAQ and VSS.

**Conclusion:**

Although both interventions had a significant effect on PSAQ and VSS, compared to Face Duox, the topical use of Aloe Vera cream significantly reduced scar formation after alar resection, both statistically and clinically.

## Introduction

Rhinoplasty is the fourth most common anesthetic surgery in the world (1). This procedure is the most common method for recontouring the nose and cosmetic purposes, which can be done using external and endonasal or close approaches (2).

Rhinoplasty may involve the incision of the alar base region, which could increase the risk of an obvious skin scar (3). Alar base surgery is commonly used for patients with an asymmetric nostril base, a wide alar, and a flared alar (4,5). The shape and symmetry of the alar base and nostrils play a vital role in the final aesthetic results of the operation. 

If the surgical rhinoplasty is performed well with proper surgical techniques, but the scar is not well repaired, the final aesthetic results of the operation are reduced. By adhering to technical factors, including correct planning of the location and design of the incision, precise execution, extensive multilayer closure, and appropriate postoperative care, such as using anti-scar materials, wound scarring could be minimized (6-8). An anti-scar agent can influence scar formation and subsequently decrease noticeable alar or columellar scar. So far, various topical medications have been used to reduce scarring after surgery. There has been a great deal of interest in aloe vera. In previous studies, many properties have been mentioned about aloe vera, and one of the most important of its application is for wound healing (9,10).

This plant is well known for its therapeutic potential, and numerous therapeutic properties have been reported, including wound and burn healing, antifungal, anti-inflammatory, anticancer, gastro-protective, anti-ulcer, antidiabetic, antioxidant, antihyperlipidemic properties (10). These numerous properties have made this material also commercially valuable. It is produced in various forms, such as aloe vera juice, concentrate, and powder and is generally used in pharmaceuticals, foods, and cosmetics. Aloe vera is applied for wound dressing in different forms, such as electrospun fibers, hydrogels, membranes, sponges, particles, and fabrics (9). Many studies have been done on using aloe vera in wound healing, but to the best of our knowledge, fewer studies have been done to assess this material's influence on reducing alar surgical site scars. Therefore, the purpose of the present study was to determine the effect of a newly developed aloe vera cream on the healing of alar wounds after rhinoplasty.

## Materials and Methods

*Trial design*

This study was a randomized, double-arm, parallel-group, double-blind trial approved by Shiraz University of Medical Sciences' Local Medical Ethics Committee (approval ethics number: IR.SUMS.MED.REC.1400.221) and registered at the Iranian Registry of Clinical Trials (IRCT20170704034897N3). 

*Participants*

The study was done at Shiraz Motahari ENT clinic, Khalili, Ghadir, and Shahid Dastgheib hospitals from June 2021 to February 2022. This study included all males and females between the ages of 18 and 60 who had undergone rhinoplasty with alar removal and wished to participate. On the other hand, exclusion criteria include participants who have a history of previous rhinoplasty or nose trauma diseases that impair wound healing, such as connective tissue diseases or diabetes, a history of using other wound healing drugs during the study, a significant postoperative hematoma, a skin disease, and previous scar on the site of alar. The skin type of the patients was the dominant type of the Iranian race (Fitzpatrick classification type 3 and 4).

*Interventions*

An expert ENT specialist performed all rhinoplasty surgeries, and an alar wedge resection was done for them. Therefore, the surgical technique was the same for both groups. The columellar and alar incision is closed in two layers using deep 6/0 chromic catgut sutures and skin 6/0 nylon sutures. The skin sutures were removed on the seventh day after the operation. Patients were randomly assigned to receive either aloe vera cream (n = 31) or Face Doux cream (n = 29) twice a day for one month as the intervention and comparison group, respectively. In the intervention group (group A), aloe Vera cream was used, while in the comparison group (group B), Face Doux cream (Wound healing cream with good performance and available in the market) was used. The test drug consisted of aloe vera cream prepared by a pharmacist (Dr. Zarshenas, professor at the School of Pharmacy of Shiraz University of Medical Sciences). The ala base scar was evaluated two weeks, two months, and six months after surgery. In addition, we performed the hypersensitivity control test by using the cream 48 hours before using the cream in the main area (nasal ala), in a circle with a diameter of 2 cm on the back of the patient's arm, and the immediate and delayed signs of hypersensitivity were assessed. We checked itching, burning and redness, and if these signs appeared, we excluded the patient from the study.

*Preparation of the aloe vera cream*

The cream was an emulsion and composed of two Aqueous and oily phases. The following percentages are based on weight. The oily phase consists of the following components: stearyl alcohol was 14%, macrogol was 14%, glycerin Sweet was 12%, Almond Oil was 4%, Butylated hydroxytoluene was 15% (Butylated hydroxytoluene used as an antioxidant).

We Mixed these ingredients and liquefied them by applying a temperature of 70 ° C. The Aqueous phase is composed of the following components: ‌‌‌Aloe vera powder was 2% to methylparaben, 0.05% to propyl paraben Water at the rate of 6.53% (paraben methyl and paraben propyl have been used as protection against bacterial and fungal agents). We Mixed the above components at 70 ° C too. Then we mixed the aqueous and oily phases immediately with a mixer until an emulsion was formed.

*Outcomes*

The primary outcome measure was the wound scar status two weeks, two months, and six months after the initial surgery. It was assessed by two Questionnaires, including the mean Patient Scar Assessment Questionnaire (PSAQ) and Vancouver Scar Scale (VSS) that validated and culturally adopted to Persian (11-13). PSAQ was a self-administered questionnaire that patients filled and ranged from 7-70 scores (well healing to undesirable healing). While VSS was based on the physicians’ clinical judgment and filled by them and ranged from 0-13 scores (well healing to undesirable healing).

*Sample size*

For sample size, 30 people were obtained in each group based on a previous similar study considering the first type error equal to 0.05 and test power equal to 0.8 using NCSS software and the following formula and according to the mean value and standard deviation of the erythema index of scars in the two study groups of the previous study 14 which were 617.5 ± 10 and 627.7 ± 14.9, respectively.

$n_{1}=n_{2}=\frac{{{2\sigma^{2}(Z}_{1-\alpha /2}+Z_{1-\beta )}}^{2}}{{\left(\mu_{1}-\mu_{2}\right)}^{2}}$

*Randomization*

Randomization of patients into two parallel treatment groups will be done based on the list obtained from the permuted block method. The simple random allocation was performed to allocate patients into the two groups via the table of random numbers. A statistician generated the randomized list by using NCSS (statistical software). According to the randomization list, one ENT specialist visited all the patients and assigned them to the drug or control group.

*Blinding*

A blinded treatment allocation was used for the participants, the clinical outcome assessors (a dermatologist and an independent ENT senior resident) who examined patients following surgery and gave them cream, and the statistician who analyzed the data. This was until statistical analysis was complete. Both aloe vera and Face Doux creams were placed in exactly the same containers.

*Statistical methods*

Descriptive statistics were reported as mean and SD or frequency (%), and between-group comparisons was performed using an independent T-test. Also, repeated measurement analysis was used to assess the intervention effect in each group. SPSS 21.0 was used for all statistical analyses, and P<0.05 was considered statistically significant.

## Results

*Baseline data*

**Fig. 1:**
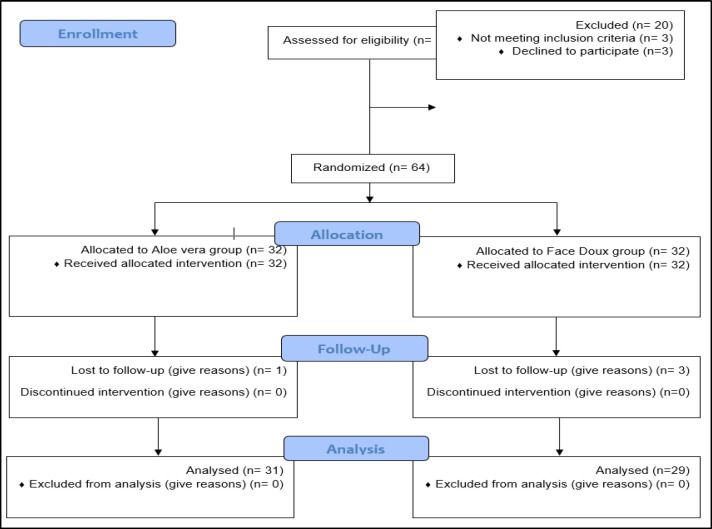
CONSORT diagram of the participants

In this study, 31 patients in group A and 29 in group B were included (Figure 1) in the final analysis, with a mean age of 27.9±7.4 years. The mean age in group A was 27.6± 7.5 years, and in group B was 28.3± 7.4 years without a statistically significant difference (P=0.71). Also, most patients in both groups were female (87.1% versus 86.2%, P=0.99).

*Outcomes and estimation*

The mean Patient Scar Assessment Questionnaire (PSAQ) was significantly lower in group A after two weeks (26.9 versus 31.5, P<0.001), after two months (15.7 versus 19.6, P=0.04) and six months follow up (8.8 versus 11.8, P=0.005). The mean Vancouver Scar Scale (VSS) was significantly lower in group A after two weeks (5.6 versus 7.1, P=0.001), after two months (3.5 versus 4.9, P=0.002), and six months (1.2 versus 2.7, P<0.001).

**Fig 2:**
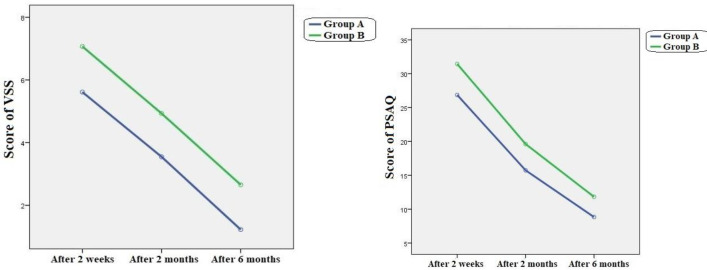
The time trend of PSAQ and VSS score changes in each group

Repeated measurement analysis showed that both interventions significantly affected PSAQ and VSS (Table 1 and Figure 2).

**Table 1:** Comparison of between and within group scores

**Variables**	**Groups**	2 weeks**		**2 months** ^#^		**6 months** ^#^		**Time effect** P-value*	**Group effect** P-value*	**Time group effect** P-value*
		Mean (SD)	Cohens d (%95 CI)	Mean (SD)	Cohens d (%95 CI)	Mean (SD)	Cohens d (%95 CI)			
Patient Scar Assessment Questionnaire	A	26.9±4.5	-0.96 (-1.50 to-0.42)	15.7±3.3	-1.11(-1.66 To -0.57)	8.8± 2.0	-1.21 (-1.76 to-0.66)	<0.001	<0.001	0.22
	B	31.5± 5.1		19.6± 3.7		11.8± 2.9		<0.001		
Between group comparison P-value		<0.001		0.04		0.005				
		Mean (SD)	Cohens d (CI)	Mean (SD)	Cohens d (CI)	Mean (SD)	Cohens d (CI)			
Vancouver Scar Scale	A	5.6± 1.5	-0.97-1.50 to -0.43	3.5± 0.9	-1.40 -1.96 to-0.83	1.2± 0.9	-1.50-2.10 to-.092	<0.001	<0.001	0.93
	B	7.1± 1.6		4.9± 1.1		2.7± 1.1		<0.001		
Between group comparison P-value		0.001		0.002		<0.001				
* Repeated measurement analysis ** Independent T-test # Analysis of covariance (considering pre-test as covariate)										

## Discussion

We found that compared to group B, the mean Patient Scar Assessment Questionnaire (PSAQ) and Vancouver Scar Scale (VSS) were significantly lower in group A after two weeks, after two months, and after six months of follow-up. Furthermore, repeated measurement analysis showed that both interventions had statistically and clinically significant effects on PSAQ and VSS, and these effects increased over time. Because rhinoplasty is performed mainly to increase the beauty, it is very important to use methods to improve the healing of the surgical site scar as much as possible. Alar soft-tissue techniques are often necessary to obtain acceptable outcomes and superior results when we do rhinoplasty (15). An effective columellar scar results from accurately planning and executing the incision, meticulously securing the wound, and maintaining good postoperative care (6). 

In this regard, in our study, the senior author performed all surgeries in both groups, so the alar wedge resection technique was the same. 

Besides using proper techniques for alar base reduction from the past until now, finding methods to improve the healing of the scars of surgical wounds, especially in cosmetic surgery, has been one of the topics that researchers have paid much attention to, and so far, different methods have been used with conflicting results. Some studies showed no effect, for example, the scar of the alar base incision after rhinoplasty was unaffected by fractional CO_2_ laser treatment, according to Fallahi et al. (16). In addition, Yahyavi et al. indicated that isotretinoin caused no evident disturbance to the recovery of rhinoplastic incisions (17).

Many studies showed that aloe vera gel accelerates wound healing. Based on a study conducted by Mazarello and co-workers, acne patients treated with creams containing 20% propolis, 3% tea tree oil, and 10% aloe vera (PTAC) and erythromycin cream (ERC) were found to have reduced erythema scars, acne severity index, and the total number of lesions by PTAC (18). Our finding showed that both interventions significantly reduced the erythema of the alar resection incision after two weeks of follow-up. Using Wistar rats, Tarameshloo et al. investigated the effect of topical application of Aloe vera gel, thyroid hormone cream, and silver sulfadiazine cream on wound healing (19).

According to their findings, Aloe vera gel significantly improved re-epithelialization and angiogenesis.  Taczare et al. evaluated the influence of Aloe Vera Gel on the Dermal Wound Healing Process in Rats. According to their results, the control group had a statistically significant difference in neutrophils, macrophages, and fibroblasts, as well as wound thickness (20). They observed a significant difference between the experimental and control groups in terms of wound diameter thickness. Because of the cosmetic issues, we could not perform a biopsy of alar resection wound. Therefore, we choose other assessment instruments including Scar Assessment Questionnaire (PSAQ) score and Vancouver Scar Scale (VSS) to compare the effect of Aloe Vera versus Face Dou cream on alar incision scar after rhinoplasty. We found that although both interventions had significant effect on PSAQ and VSS, the mean PSAQ score and mean VSS were significantly lower in Aloe Vera group in compare to Face Dou group after two weeks, after two months and six months follow up. 

By computing the standardized mean difference [Cohens d (%95 CI)], we showed that in comparison to Face Dou, Aloe Vera had a strong effect on PSAQ score after two weeks (Cohens d; -.096), after two months (Cohens d; -1.11) and after six months (Cohens d; -1.21) s. As you can see, this effect progressively increased over time. Again, calculating Cohens d indicated that in comparison to Face Dou, Aloe Vera had a strong effect on VSS score after two weeks (Cohens d; -.097), after two months (Cohens d; -1.40) and after six months (Cohens d; -1.50). As it is evident, these effects progressively increased over time. (Table 1 and Figure 2). 

*Strengths*

The type of study, a randomized controlled trial, was one of this study's strengths. Also, we used PSAQ and VSS questionnaires to assess the scar status, which patients and physicians filled. The advantage of using these two questionnaires simultaneously is that both the patient's and the physician's views of wound healing have been evaluated, and possible biases have been avoided. The opinion of the physicians is important because they examine the repair with a technical and specialized view, and the patient's opinion and satisfaction are a momentous influence on their quality of life.

This study did not see any significant side effects such as skin reaction, redness and inflammation. In addition, topical cream is a noninvasive method and is a better choice, especially for cosmetic procedures, compared to invasive methods such as a laser.

*Limitation and further study*

The limitation of this study was the relatively short patient follow-up period. Work on the remaining issues is also ongoing and will be presented in future articles in light of the promising findings presented in this paper. Among other things, future work will involve the application of this cream for other aesthetic surgical sites such as blepharoplasty, otoplasty, and other cosmetic surgery in the field of the head and neck. 

## Conclusion

Although both interventions had a significant effect on PSAQ and VSS, compared to Face Duo, the topical use of Aloe Vera cream significantly reduced scar formation after alar resection, both statistically and clinically.

Ethics approval

This study was performed in line with the principles of the Declaration of Helsinki. Approval was granted by the Ethics Committee of Shiraz University of Medical Sciences (IR.SUMS.MED.REC.1400.221).
